# Perceived physical exertion in senior workers performing heavy manual tasks

**DOI:** 10.1093/annweh/wxag054

**Published:** 2026-07-07

**Authors:** Albin Stjernbrandt, Pontus Öhrner, Andreas Tornevi, Jerry Öhlin, Farhad Abtahi, Charlotte Lewis, Mikael Forsman, Viktoria Wahlström

**Affiliations:** Department of Epidemiology and Global Health, Umeå University, 901 87 Umeå, Sweden; Sports Medicine, Department of Community Medicine and Rehabilitation, Umeå University, 901 87 Umeå, Sweden; Department of Epidemiology and Global Health, Umeå University, 901 87 Umeå, Sweden; Department of Epidemiology and Global Health, Umeå University, 901 87 Umeå, Sweden; Division of Ergonomics, KTH Royal Institute of Technology, 114 28 Stockholm, Sweden; Department of Clinical Science, Intervention and Technology, Karolinska Institutet, 171 77 Stockholm, Sweden; Department of Epidemiology and Global Health, Umeå University, 901 87 Umeå, Sweden; Division of Ergonomics, KTH Royal Institute of Technology, 114 28 Stockholm, Sweden; Institute of Environmental Medicine, Karolinska Institutet, 171 77 Stockholm, Sweden; Department of Epidemiology and Global Health, Umeå University, 901 87 Umeå, Sweden

**Keywords:** construction workers, kitchen workers, cleaners, assistant nurses, occupational physical activity, prolonged working life

## Abstract

With an aging workforce and longer working lives, staying in physically demanding occupations is increasingly challenging, and the factors influencing perceived exertion are still poorly understood. Also, self-rated exertion may not correlate strongly with physiological measurements of physical strain at work, such as heart rate. Our study aimed to describe the physical strain experienced by senior workers in manual occupations using Borg's Rating of Perceived Exertion (RPE) scale and to explore associations between personal variables such as heart rate during work, age, physical fitness, and RPE ratings. We recruited workers aged 50 yr or older in manual occupations and collected data using questionnaires, physical examinations, field heart rate monitoring, and RPE ratings during work. Field heart rate monitoring was conducted using a chest band with a pulse sensor that transmitted data to a mobile application, and the first 6 h of recordings were used in subsequent analyses. Subjects were asked to simultaneously complete a diary at the end of each shift, rating the overall physical exertion for that day using the RPE scale, ranging from 6 (rest) to 20 (maximal physical exertion). Correlation between variables was determined using Spearman's rank correlation coefficient (*r*). A linear mixed model was used to investigate associations between personal variables and RPE ratings. The sample consisted of 126 subjects, aged 50 to 67 yr, of whom 76 (60.3%) were females. The participants were employed as construction workers, kitchen workers, cleaners, or assistant nurses. They rated perceived physical exertion on the RPE scale once daily in one to three workdays, resulting in a total of 250 workdays. The mean (SD) RPE rating was 12.1 (2.1). When stratified by sex, the mean (SD) RPE rating was 12.1 (2.2) among females and 12.0 (1.8) among males (*P* = 0.669). When stratified by occupational group, the mean (SD) was 11.9 (1.9) for construction workers, 12.1 (2.0) for kitchen workers, 12.4 (1.6) for cleaners, and 11.8 (2.8) for assistant nurses (*P* = 0.139 to 0.815). In total, 298 heart rate (HR) recordings were obtained during work with a mean (SD) HR of 89.4 (11.4) beats/min (range 54 to 125). There was a significant bivariate correlation between the RPE ratings and mean HR for the entire study population (*r* = 0.14; *P* = 0.025) as well as in the group of construction workers (*r* = 0.23; *P* = 0.042), but not for any other occupational groups (*r* = 0.04 to 0.25; *P* = 0.096 to 0.752). In the linear mixed model, using the RPE ratings as the dependent variable, the statistically significant (*P* < 0.05) estimated coefficients (SE) were as follows: mean heart rate (beats/min), 0.03 (0.01); very poor sleep quality, 3.38 (0.93); and high psychological stress, 2.62 (0.71). Age, body mass index, theoretical maximum oxygen consumption, and chronic pain ratings were not significantly associated with the RPE ratings. We conclude that senior workers in manual occupations reported that their work was *somewhat hard* in terms of physical exertion. Our study also indicates associations between mean heart rate, sleep quality, psychological stress, and RPE ratings.

What’s Important About This Paper? The retirement age is being raised in many countries, and workers in heavy manual jobs are expected to maintain performance into older age. Little is known about senior manual workers and their perceived rate of physical exertion or what factors influence their capacity. This study found that mean heart rate, sleep quality, and psychological stress affect self-reported physical exertion. Sleep quality and psychological stress would be suitable targets for future interventions to support the health of senior manual workers.

## Introduction

Physical demands are high in many manual occupations, and 43% of the Swedish workforce reports a strenuous job (Swedish Work Environment Authority 2022). However, it has proven challenging to find valid and straightforward measures of occupational physical activity (OPA) using questionnaires (Kwak et al. 2011). The strain in occupations with high OPA can be interpreted in terms of both the musculoskeletal and cardiovascular systems, and strenuous work can predispose workers not only to musculoskeletal disorders but also to an increased risk of major adverse cardiovascular events and death (Coenen et al. 2018, 2024; Holtermann et al. 2021). Moreover, occupations with a high OPA and related adverse health effects can have a considerable socio-economic impact, affecting not only workers and employers but also society in general. A study in Denmark found that one in four senior employees in physically demanding occupations reported that poor physical health and the inability to perform their work tasks would eventually force them to leave the labor market early (Andersen et al. 2020). Analyses of Swedish official statistics have shown that blue-collar workers often withdraw from work before the general retirement age (ie before 65 yr; Kadefors et al. 2018). It has been previously shown that the relative workload of a given task is lower for individuals with higher physical capacity or fitness (Haff and Triplett 2015). As part of the natural aging process, physical capacity declines, most notably in terms of aerobic capacity (Boss and Seegmiller 1981; Ilmarinen et al. 1991; Merkus et al. 2019). The general retirement age is being raised in many countries as the population structure changes (The Organization for Economic Co-operation and Development 2023). While the overall life expectancy increases, the number of disease-free years is decreasing. Consequently, if the physical demands at work are maintained with increasing age, more employees in physically demanding occupations will be expected to work with a higher *relative* cardiovascular workload (Merkus et al. 2019).

To promote a sustainable environment for older employees, it is essential to be able to measure the specific occupational physical demands they face. This calls for reliable, valid, and easy-to-use methods for assessing OPA to perform risk assessments and take preventive actions. Borg's Rating of Perceived Exertion (RPE) scale (Borg et al. 1985) is a simple method used in occupational medicine to measure specific perceived physical exertion (Johansson and Ljunggren 1989; Balogh et al. 2004; Jakobsen et al. 2014; Korshøj et al. 2022). The RPE scale is closely related to exercise intensity or cardiac load during steady-state work, with previous research demonstrating a moderate to high correlation between RPE ratings and heart rate (HR), as well as several other physiological variables (Borg 1982; Robertson 1982; Chen et al. 2002). Moreover, ratings on the RPE scale have been directly translated into the associated heart rate or percentage of heart rate reserve (%HRR) in an occupational setting (Korshøj et al. 2022), and correlated with physiological variables as if the RPE scale intended to function interchangeably with these (Balogh et al. 2004; Scherr et al. 2013; Jakobsen et al. 2014). Often, the RPE scale ranging from 6 to 20 is used to extrapolate a rough estimate of HR by multiplying the RPE rating by 10. However, this scale was not originally intended to be used interchangeably with physiological variables to measure intensity or load (Borg 1998). Moreover, the ratings can also potentially be influenced by many other factors than physical strain, such as age, type of exercise, somatic symptoms (eg pain or fatigue), psychological factors (eg stress or anxiety), environmental factors (eg noise or temperature), and rating behavior (ie individual differences in how to interpret the scale; Borg 1982). In fact, multiplying the RPE rating by 10 will often not equal heart rate for healthy people of a given age (Pandolf 1983), and several studies have questioned whether HR is a prominent cue for perceived physical exertion (Davies and Sargeant 1979; Sjöberg et al. 1979; Martin 1981; Robertson 1982). For intermittent or rapidly changing work tasks, even less is known about the suitability of the RPE scale. Additionally, in an occupational setting, compared to a controlled laboratory environment, several external factors are likely to influence the perception of physical exertion. Therefore, there is a need to further explore the extent to which different psychological and physical variables explain ratings on the RPE scale under normal working conditions, to better understand how to interpret the ratings when used to measure perceived occupational load. Additionally, it is crucial to enhance knowledge of the factors that contribute to workers’ experiences of strenuous work, thereby creating a sustainable work environment that enables workers to continue performing at higher ages.

Our study aimed to describe the physical strain experienced by senior workers in manual occupations using Borg's RPE scale and to explore the associations between personal variables such as HR during work, age, physical fitness, and RPE scale ratings.

## Materials and methods

Using cross-sectional data from questionnaires, health examinations, and device-measured HR data, our study was designed to analyze potential associations between physiological response variables (HR or %HRR), demographics (age, body composition, and physical fitness), and health conditions (sleep quality, psychological stress, and chronic pain ratings) in relation to RPE ratings.

### Participants and procedures

Our study was based on data collected in the High Physical Exposure (HIPE) research project, which was launched to develop a method for technically measuring physical workload and to investigate the physical demands among professionals aged 50 yr or older in physically demanding jobs (Wahlström et al. 2025). The data used in our study were collected between August 2021 and April 2023. The study protocol was approved by the Swedish Ethical Review Authority (DNR 2020-01927, 2020-05850, and 2021-03400), and all participants provided written informed consent to participate.

The study included construction workers, kitchen workers, cleaners, and assistant nurses. The inclusion criteria were age 50 yr, an employment rate ≥ 50%, and full-day work hours to enable field measurements. A total of 129 subjects were initially recruited; however, three were excluded after consultation with a cardiologist due to heart disease that compromised the validity of HR measurements (Sammito et al. 2024); one due to current atrial fibrillation, one because of congestive heart failure with bradycardia, and one due to severe ischemic heart disease (Wahlström et al. 2025). Hence, 126 subjects were included in the analyses.

### Questionnaire

Prior to the field measurements, all participants answered a questionnaire that contained questions on age, sex, employment status, and occupational title. Sleep quality was investigated using a validated questionnaire (Gustafsson et al. 2006). In detail, sleep was assessed through the survey item: “How is your sleep in general?” with five response alternatives ranging from “Very good” to “Very poor.” The presence of chronic pain was investigated using the revised Graded Chronic Pain Scale (GCPS-R; Von Korff et al. 2020). GCPS-R is a validated scale for chronic pain and its interference with daily activities, comprising five questions. A total score for the five questions was calculated and classified into four categories: “no pain,” “mild chronic pain,” “bothersome chronic pain,” and “high impact chronic pain.”

### Physical examination

The subjects underwent a standardized health examination performed by research nurses at the Clinical Research Center at the University Hospital of Umeå. Participants were asked to refrain from nicotine, coffee, and tea in the morning before the health examinations, and to avoid heavy exercise and stress on the day before the examinations. The participants’ heights were measured without shoes on using a digital stadiometer (Seca 264, Hamburg, Germany) with an accuracy of ±0.1 cm. Weight was measured using a digital scale (Seca 861, Hamburg, Germany) with a ±0.1 kg accuracy while wearing light clothing or underwear. From these data, body mass index (BMI) was calculated and used in the analyses. In the material description, BMI was categorized according to clinically used thresholds for underweight, overweight, and obesity (World Health Organization 1995). Waist circumference was measured with a measuring tape placed midway between the top of the iliac crest and the lower edge of the last palpable rib, with ±0.5 cm accuracy. Blood pressure was measured on the right upper arm at heart level using a blood pressure monitor (Omron M6, Kyoto, Japan) with an accuracy of ±0.5 mmHg when the participant had been resting in a sitting position for 5 min. The mean of two blood pressure measurements was recorded. The resting HR was measured with a Polar HR 9 monitor (Polar Electro Oy, Kempele, Finland), as the lowest recorded HR during 10 min of lying rest. To estimate the theoretical maximum oxygen consumption (VO_2_max), an aerobic physical capacity test was performed on a cycle ergometer (Ergomedic 828 E, Monark AB, Varberg, Sweden) using the Ekblom-Bak submaximal test procedure (Ekblom-Bak et al. 2014).

### Field measurements

Following the health examinations, field measurements of HR were conducted over three workdays using a chest band with a pulse sensor (Suunto Movesense, Helsinki, Finland) that transmitted data via Bluetooth to a mobile application (Wergonic AB, Stockholm, Sweden). The workers were instructed to wear the pulse sensor for the entire workday, regardless of activity, and the first 6 h of recordings were used in subsequent analyses. The 6-h duration was selected to balance between maximizing the measurement period and retaining a sufficient number of valid measurement days for analysis. The %HRR was calculated using the following formula: %HRR = (HR_mean_ − HR_min_)/(HR_max_ − HR_min_) × 100 (Tanaka et al. 2001; Dias et al. 2023). Maximum HR was calculated as follows: 208 − 0.7 × age (Tanaka et al. 2001). For the minimum HR, the lowest of the following was used: the resting HR measured at the health examination or the lowest HR from the working day measurement, with a 15-s running window. Further details on data processing have been previously published (Wahlström et al. 2025). During the field measurements, subjects were asked to complete a day-to-day diary at the end of each shift, rating the overall physical exertion for that day using the RPE scale, ranging from 6 (rest) to 20 (maximal physical exertion; Borg 1982). The diary also included items on psychological stress during the workday, based on Karasek and Theorell's concepts and the original works of the Stress Research Institute at Stockholm University, Sweden (Karasek and Theorell 1990). The subjects were asked to answer the question: “How have you felt during the workday?” and responses were graded on a nine-point scale ranging from “Very low stress (have felt very relaxed and calm)” to “Very high stress (have felt very tensed and pressured—at the limit of what I can cope with).” While general sleep quality and the presence of chronic pain were assessed only once, the RPE ratings and psychological stress evaluations were repeated daily during the field measurements.

### Statistical analysis

Numerical data were normally distributed and described as means and standard deviations, while categorical data were presented as numbers and valid percentages. Missing data were excluded from analyses. Mean values for repeated RPE ratings and HR recordings during work were aggregated. Correlation between scales was determined using Spearman's rank correlation coefficient (*r*). Associations between numerical variables (mean HR or %HRR, age, BMI, and VO_2_max), categorial variables (sleep quality, psychological stress, and chronic pain rating), and the dependent variable (RPE ratings) were analyzed using linear mixed models with a random intercept to account for multiple ratings from the same subjects and expressed as estimated coefficients (*B*) with standard error (SE). The independent variables included in the model were determined a priori to be relevant to the aim of the study, based on discussions within the author group, including ergonomists, a physiologist, a statistician, an engineer, and an occupational physician. First, we wanted to include relevant physiological response variables and chose HR or %HRR. Since the study focused on older workers with varying levels of physical fitness, we included age, BMI, and VO_2_max in the model. Finally, we wanted to investigate common personal health conditions in older age, such as insomnia, stress, and chronic pain. However, the number of independent variables was limited by the sample size and potential collinearity.

## Results

### Study population

The study included 126 subjects, of whom 76 (60.3%) were female, and 50 were male, with a mean (SD) age of 57 (4) yr for both sexes. They worked as construction workers (carpenters, painters, concrete workers, floor layers, and tilers), commercial kitchen workers (chefs and kitchen assistants), cleaners, or assistant nurses. Detailed descriptive data are presented in Table 1.

**Table 1:** Participant demographics and self-reported health conditions.

		All subjects	Construction workers	Kitchen workers	Cleaners	Assistant nurses
Variable	Category	*N* (%)	*N* (%)	*N* (%)	*N* (%)	*N* (%)
Study participants	…	126	35	36	29	26
Age (yr)	50 to 55	46 (36.5)	15 (42.9)	11 (30.6)	9 (31.0)	11 (42.3)
	56 to 61	63 (50.0)	17 (48.6)	18 (50.0)	14 (48.3)	14 (53.8)
	62 to 67	17 (13.5)	3 (8.6)	7 (19.4)	6 (20.7)	1 (3.8)
Sex	Female	76 (60.3)	0 (0.0)	27 (75.0)	25 (86.2)	24 (92.3)
	Male	50 (39.7)	35 (100.0)	9 (25.0)	4 (13.8)	2 (7.7)
Body mass index	Underweight	1 (0.8)	0 (0.0)	0 (0.0)	1 (3.4)	0 (0.0)
	Normal weight	46 (36.5)	12 (34.3)	15 (41.7)	8 (27.6)	11 (42.3)
	Overweight	55 (43.7)	21 (60.0)	14 (38.9)	11 (37.9)	9 (34.6)
	Obese	24 (19.0)	2 (5.7)	7 (19.4)	9 (31.0)	6 (23.1)
Smoking	Current	6 (4.8)	2 (5.7)	2 (5.6)	0 (0.0)	2 (8.0)
	Former	33 (26.4)	5 (14.3)	9 (25.0)	12 (41.4)	7 (28.0)
	Never	86 (68.8)	28 (80.0)	25 (69.4)	17 (58.6)	16 (64.0)
Oral moist tobacco use	Current	22 (17.6)	10 (28.6)	5 (13.9)	4 (13.8)	3 (12.0)
	Former	16 (12.8)	7 (20.0)	2 (5.6)	3 (10.3)	4 (16.0)
	Never	87 (69.6)	18 (51.4)	29 (80.6)	22 (75.9)	18 (72.0)
Diseases	Asthma	12 (9.5)	1 (2.9)	5 (13.9)	3 (10.3)	3 (11.5)
	Hypertension	42 (33.3)	12 (34.3)	13 (36.1)	10 (34.5)	7 (26.9)
	Atrial fibrillation	2 (1.6)	0 (0.0)	0 (0.0)	1 (3.4)	1 (3.8)
	Other heart disease	8 (6.3)	1 (2.9)	4 (11.1)	1 (3.4)	2 (7.7)
	Diabetes	10 (7.9)	1 (2.9)	4 (11.1)	4 (13.8)	1 (3.8)
Sleep quality	Very good	22 (17.6)	8 (22.9)	6 (16.7)	6 (20.7)	2 (7.7)
	Good	50 (40.0)	16 (45.7)	17 (47.2)	9 (31.0)	8 (32.0)
	Neither good nor bad	28 (22.4)	9 (25.7)	7 (19.4)	6 (20.7)	6 (24.0)
	Poor	22 (17.6)	2 (5.7)	5 (13.9)	7 (24.1)	8 (32.0)
	Very poor	3 (2.4)	0 (0.0)	1 (2.8)	1 (3.4)	1 (4.0)
Psychological stress^a^	1 (Very low)	6 (5.5)	1 (3.0)	3 (9.1)	1 (4.2)	1 (5.0)
	2	10 (9.1)	2 (6.1)	4 (12.1)	2 (8.3)	2 (10.0)
	3 (Low)	28 (25.5)	8 (24.2)	6 (18.2)	8 (33.3)	6 (30.0)
	4	11 (10.0)	0 (0.0)	3 (9.1)	5 (20.8)	3 (15.0)
	5 (Neither high nor low)	38 (34.5)	19 (57.6)	10 (30.3)	6 (25.0)	3 (15.0)
	6	4 (3.6)	0 (0.0)	3 (9.1)	1 (4.2)	0 (0.0)
	7 (High)	13 (11.8)	3 (9.1)	4 (12.1)	1 (4.2)	5 (25.0)
	8	0 (0.0)	0 (0.0)	0 (0.0)	0 (0.0)	0 (0.0)
	9 (Very high)	0 (0.0)	0 (0.0)	0 (0.0)	0 (0.0)	0 (0.0)
Chronic pain	No chronic pain	72 (56.0)	17 (48.6)	24 (66.7)	16 (55.2)	13 (52.0)
	Mild chronic pain	17 (13.6)	4 (11.4)	4 (11.1)	4 (13.8)	5 (20.0)
	Bothersome chronic pain	8 (6.4)	3 (8.6)	3 (8.3)	1 (3.4)	1 (4.0)
	High impact chronic pain	30 (24.0)	11 (31.4)	5 (13.9)	8 (27.6)	6 (24.0)

^a^Data from the first day of measurements.

### Ratings of perceived physical exertion at work

The study participants rated perceived physical exertion on the RPE scale once daily in one to three workdays, resulting in a total of 250 workdays reported by 104 subjects. The ratings ranged from 7 to 20, with a mean (SD) RPE rating of 12.1 (2.1). When stratified by sex, the mean (SD) RPE rating was 12.1 (2.2) among females and 12.0 (1.8) among males (*P* = 0.669). When stratified by occupational group, the mean (SD) was 11.9 (1.9) for construction workers, 12.1 (2.0) for kitchen workers, 12.4 (1.6) for cleaners, and 11.8 (2.8) for assistant nurses (*P* = 0.139 to 0.815). When analyzing ratings from the first workday only (*n* = 88), the mean (SD) RPE rating was 11.7 (2.1).

### Measured heart rate at work

In total, 298 HR recordings were obtained during work on 120 subjects with a mean (SD) duration of 358 (10) min and a mean (SD) HR of 89.4 (11.4) beats/min (range 54 to 125), representing a mean (SD) HRR of 25.0% (7.6). Most missing data arose from real-time Bluetooth transmission failure or insufficient recording duration. Mean, minimum, and maximum HR, separated by sex and occupational group, are presented in Table 2. When only analyzing HR recordings from the first workday (*n* = 103), the mean (SD) HR was 89.8 (11.7) beats/min. There was a significant bivariate correlation between the RPE ratings and mean HR for the study population as a whole (*r* = 0.14; *P* = 0.025) as well as in the group of construction workers (*r* = 0.23; *P* = 0.042), but not for any of the other occupational groups (*r* = 0.04 to 0.25; *P* = 0.096 to 0.752). There was no significant association between age and measured heart rate at work, neither expressed as mean HR (*B* 0.012; SE 0.16; *P* = 0.478) nor as %HRR (*B* 0.15; SE 0.11; *P* = 0.150).

**Table 2:** Descriptive data from the physical examinations and heart rate measurements during work.

	All subjects	Construction workers	Kitchen workers	Cleaners	Assistant nurses
	Mean (SD)	Mean (SD)	Mean (SD)	Mean (SD)	Mean (SD)
**Physical examinations (*N* = 126 subjects)**					
Resting HR (beats/min)	68.4 (10.6)	65.2 (10.0)	68.7 (9.5)	70.7 (12.2)	69.8 (10.3)
Systolic BP (mmHg)	137.4 (18.3)	141.6 (17.3)	136.8 (20.1)	135.6 (19.3)	134.7 (15.6)
Diastolic BP (mmHg)	84.6 (9.8)	86.2 (9.9)	82.3 (10.1)	84.1 (10.5)	86.3 (8.3)
Height (cm)	168.1 (9.3)	177.4 (6.2)	165.2 (8.5)	164.5 (7.5)	163.4 (6.5)
Weight (kg)	76.0 (14.2)	82.5 (10.4)	73.0 (13.0)	75.7 (15.5)	71.6 (16.4)
BMI (kg/m^2^)	26.8 (4.4)	26.1 (2.4)	26.7 (3.9)	28.0 (5.4)	26.7 (5.5)
Waist circumference females (cm)	90.2 (13.4)	−^a^	86.7 (10.7)	94.6 (15.0)	89.6 (13.4)
Waist circumference males (cm)	97.0 (10.4)	95.3 (8.7)	102.5 (8.0)	96.3 (18.9)	104.0 (25.5)
VO_2_max females (ml/kg/min)	30.1 (6.7)	−^a^	30.2 (6.2)	29.4 (8.0)	30.8 (5.9)
VO_2_max males (ml/kg/min)	38.1 (7.8)	39.1 (6.6)	35.7 (6.6)	38.4 (12.5)	31.2 (22.2)
**HR measurements during work (*n* = 120 subjects, *n* = 298 recordings)**					
Mean HR (beats/min)	89.4 (11.4)	90.6 (11.9)	87.7 (11.9)	91.1 (11.1)	88.1 (9.9)
Minimum HR (beats/min)	58.5 (9.2)	57.7 (7.9)	57.0 (9.4)	60.3 (9.2)	59.6 (10.2)
Maximum HR (beats/min)	127.9 (13.9)	131.9 (15.5)	124.6 (15.0)	128.4 (12.0)	127.0 (10.6)
Mean HRR (%)	25.0 (7.6)	27.1 (8.2)	23.0 (7.2)	26.3 (7.7)	23.5 (5.9)

BMI: body mass index, BP: blood pressure, HR: heart rate, HRR: heart rate reserve, VO_2_max: estimated maximum oxygen consumption.

^a^There were no female construction workers in the study sample.

### Factors influencing RPE ratings

There were significant associations between mean HR, sleep quality, and psychological stress in relation to the RPE ratings in the linear mixed model (Table 3). Both reporting very poor sleep and high psychological stress increased the RPE ratings by about three points. Age, BMI, VO_2_max, and chronic pain ratings were not significantly associated with the RPE ratings. The largest increase in explained variance (pseudo-*R*^2^) was observed after the inclusion of personal factors. In sex-stratified analyses, very poor sleep quality and psychological stress were associated with RPE ratings in females, while mean HR and psychological stress were associated with RPE ratings in males (Tables S1 and S2). The model was also repeated using %HRR instead of mean heart rate, but there was no significant association between this measure of aerobic work intensity and RPE ratings (Table S3).

**Table 3:** Stepwise linear mixed model for Borg's Rating of Perceived Exertion scale.

			95% Confidence interval				
Variable	Categories	Estimated coefficient (β)	Lower	Upper	Standard error	*P* value	Pseudo-*R*^2^
**Step 1: Only physiological variables**							0.033
Mean HR (beats/min)	…	0.03	0.002	0.05	0.01	0.033	
Age (yr)	…	0.02	−0.05	0.09	0.03	0.588	
BMI (kg/m^2^)	…	0.06	−0.01	0.14	0.04	0.108	
VO_2_max (ml/kg/min)	…	0.03	−0.01	0.07	0.02	0.102	
**Step 2: Adding personal variables**							0.248
Mean HR (beats/min)	…	0.03	0.003	0.05	0.01	0.027	
Age (yr)	…	−0.02	−0.09	0.05	0.03	0.553	
BMI (kg/m^2^)	…	0.04	−0.04	0.11	0.04	0.357	
VO_2_max (ml/kg/min)	…	0.03	−0.01	0.07	0.02	0.174	
Sleep quality	Very good	Reference	…	…	…	…	
	Good	0.26	−0.50	1.01	0.39	0.507	
	Neither good nor bad	0.47	−0.34	1.28	0.41	0.254	
	Poor	0.05	−0.83	0.92	0.44	0.918	
	Very poor	3.38	1.55	5.22	0.93	<0.001	
Psychological stress	1 (Very low)	Reference	…	…	…	…	
	2	0.96	−0.66	2.58	0.82	0.243	
	3 (Low)	1.09	0.16	2.02	0.47	0.022	
	4	1.91	0.84	2.98	0.54	<0.001	
	5 (Neither high nor low)	1.92	1.00	2.85	0.47	<0.001	
	6	2.22	1.14	3.31	0.55	<0.001	
	7 (High)	2.62	1.23	4.01	0.71	<0.001	
Global chronic pain scale—revised	No chronic pain	Reference	…	…	…	…	
	Mild chronic pain	−0.42	−1.22	0.37	0.40	0.294	
	Bothersome chronic pain	0.64	−0.43	1.71	0.54	0.241	
	High impact chronic pain	−0.04	−0.63	0.55	0.30	0.885	

BMI: body mass index, HR: heart rate, VO_2_max: estimated maximum oxygen consumption.

## Discussion

### Main findings

In our study, senior workers in manual occupations reported their work to be *somewhat hard* in terms of physical exertion, with a mean RPE rating of about 12 on Borg's scale, ranging from 6 to 20, although with substantial intra-individual variation. There was neither significant difference in RPE ratings between female and male workers nor between occupational groups. The bivariate correlation between the RPE ratings and mean HR was weak (*r* = 0.14) for the study population as a whole and was mainly driven by the correlation among construction workers. This finding implies that self-reported ratings of occupational physical exertion are not easily compared with physiological measures of cardiac load. In the linear mixed model, significant associations were found between mean HR, very poor sleep quality, and high psychological stress in relation to the RPE ratings. In contrast, age, BMI, VO_2_max, and chronic pain ratings were not significantly associated with the ratings of perceived physical exertion.

### Interpretation

We believe that our findings are consistent with Borg's original conceptualization of perceived exertion as an integrative psychophysiological construct, arising from both central and peripheral cues, rather than a direct correlate of cardiovascular strain (Borg 1982). This framework may also account for the relatively weak association observed between RPE and mean heart rate (*r* = 0.14) in our study. Consequently, when using RPE as a measure of occupational physical demands, researchers and practitioners should be aware that there are multifactorial and highly contextual influences on these ratings and consider psychological and recovery-related factors alongside traditional biomechanical and cardiovascular assessments. This is further emphasized by the fact that the largest increase in explained variance in the stepwise linear mixed model was observed after inclusion of personal rather than physiological variables.

The group of construction workers in our study differed from the other study participants, as they were all male, which was reflected in both anthropometric measurements and VO_2_max. Among assistant nurses, there was only one male subject. In other descriptive terms, the occupational groups included in the study were roughly comparable, as indicated in Table 1. The mean (SD) VO_2_max of the construction workers in our study of 39.1 (6.6) ml/kg/min was higher than what was found in a previous study on male construction workers ≥45 yr (*n* = 23) who had a mean (SD) VO_2_max of 33.1 (5.2) ml/kg/min, using a similar submaximal exercise test (Merkus et al. 2019). Furthermore, they found associations neither between age and HR (expressed as %HRR) nor age and OPA (assessed using accelerometry; Merkus et al. 2019). This finding aligns with our study, in which neither mean HR, %HRR, nor RPE ratings were significantly associated with age. In a Swedish study on self-assessed and directly measured OPA in hospital cleaners and office workers, an RPE scale ranging from 0 to 14 was used and compared to HR measurements expressed as the percentage of HR ratio (Balogh et al. 2004). In their combined material, a significant intraclass correlation was observed between rated physical exertion and the percentage of HR ratio (*r* = 0.64; *P* < 0.001), which was not observed when analyzing the occupational groups separately. Cleaners with musculoskeletal complaints rated the perceived physical exertion about two points higher compared with asymptomatic subjects. This finding was not supported by our study, which found no effect of chronic pain on RPE ratings. Moreover, the study by Balogh et al. did not find that older cleaners (≥45 yr of age) experienced greater physical exertion than younger subjects, which is in line with our results, in which no effect of age on RPE ratings was observed. In a Danish study on workers performing manual lifting tasks (*n* = 200), of which 52 (26%) were over 50 yr of age, an RPE scale ranging from 0 to 10 was used to rate OPA (Jakobsen et al. 2014). They reported significant associations between low and high OPA, as well as between moderate and high OPA, in relation to %HRR. However, although age was used to adjust the multiple logistic regression models, no data were presented on the separate effects of aging. One interesting finding in our study was that work-related psychological stress, as measured by a simple single-item measure, had a large impact on the RPE ratings of senior workers. This is intuitive, as cognitive or emotional strain—manifested as mental fatigue or stress—has been shown to increase perceived effort during exercise, independently of physiological workload (Marcora et al. 2009). In contrast to our findings, a recent study on logistics workers found no association between psychological stress and ratings of physical exertion on a Borg scale ranging from 0 to 10 (Sala et al. 2021). However, the population in that study reported a very positive psychosocial work environment, which means that the potential adverse effects of psychological stress could not be fully discerned. The observed elevation in RPE under conditions of poor sleep quality in our study likely reflects an interaction of multiple psychophysiological mechanisms. Evidence from studies on athletic performance indicates that sleep loss disrupts central recovery processes, potentially sensitizing individuals to physical discomfort and fatigue (Fullagar et al. 2015)

When analyzing HR data in an occupational setting, several alternative strategies should be considered (Dias et al. 2023; Wahlström et al. 2025). We chose mean HR for our main analyses because we consider it a simple, intuitive way to present the average cardiac load during the workday. However, Korshøj et al. suggested that %HRR measured by worn devices should be preferred for evaluating OPA among workers with physically demanding jobs (Korshøj et al. 2022). We therefore included this measure in our data presentation but did not find a significant association with RPE ratings (Table S3).

### Limitations and strengths

There are several potential limitations to our study. To begin with, subjects who work in physically demanding occupations at higher ages may be healthier than the average worker, as those with poor health might have been forced to change occupation or leave the workforce altogether. It is also plausible that healthier subjects preferentially choose to participate in a study on physical fitness. Both mechanisms could lead to sampling bias (eg the healthy worker effect), resulting in the potential effects of aging being underestimated. The study included only 126 subjects and may not have been statistically powered to detect minor effects. There was also some internal loss of data; if all 126 subjects had provided RPE ratings and HR measurements for 3 workdays, there would have been 378 complete pairs instead of the 250 (66.1%) collected. Interestingly, there were only minor differences in mean HR and RPE ratings between the first workday and the entire measurement period. This implies that assessing these parameters for only one day could suffice. Another potential limitation is that the RPE ratings considered the entire workday, whereas the HR recordings were conducted for only about 6 h. Moreover, we estimated physical fitness using a submaximal exercise test on an ergometer. It is possible that a stronger association with RPE ratings would have been observed if the VO_2_max had been more accurately measured in a maximal exercise test using ergospirometry. Although environmental factors (eg temperature and noise) may affect RPE ratings, such data were not collected. The increase in pseudo-*R*^2^ in the stepwise linear mixed model should be interpreted with caution, as this parameter will naturally increase with the addition of multiple predictors, particularly when several categorical variables with multiple levels are included in a model with a relatively small sample. Finally, the model accounted for a relatively low proportion of the variability (pseudo-*R*^2^ = 0.248), indicating that there are other factors that influence RPE ratings that were not investigated in our study. Our study also had inherent strengths. There are very few previous studies that have investigated older workers, collecting both self-reported and field measurement data. Subjects who stated in the survey that they had atrial fibrillation or other heart disease were individually assessed by a cardiologist to determine if the HR data could be affected, resulting in the exclusion of three subjects. All remaining subjects underwent standardized physical examinations at the Clinical Research Center of our university hospital, which supports the assumption that the data were valid. The pulse measurements were also conducted systematically. For most subjects, there were several days of both pulse data and RPE ratings, reflecting varying work conditions.

### Implications

Since our study showed that both sleep quality and psychological stress affected older workers’ ratings of physical exertion in manual occupations, these would be suitable targets for interventions. For instance, employers could collaborate with occupational health services to offer psychoeducation on sleep hygiene and stress prevention. In future studies on physical exertion, these parameters would be worth including as explanatory factors.

## Conclusion

Senior workers in manual occupations reported their work to be *somewhat hard* in terms of physical exertion, with a mean RPE rating of about 12 on Borg's scale, ranging from 6 to 20. There was no major difference in ratings between female and male workers, nor between the occupational groups studied. The correlation between RPE ratings and mean HR was weak (*r* = 0.14). In the linear mixed model, significant associations were found between mean HR, sleep quality, psychological stress, and RPE ratings. Our findings support previous research indicating that RPE ratings are associated with different personal factors and not directly comparable to objective measures of cardiovascular load.

## Supplementary Material

wxag054_Supplementary_Data

## Data Availability

The data that support the findings of this study are available on request from the corresponding author. The data are not publicly available due to restrictions to protect participants’ privacy.
