# Supplementary material for: Perceived physical exertion in senior workers performing heavy manual tasks
Source: Ann Work Expo Health. 2026 Jul 7;70(5):wxag054. doi: 10.1093/annweh/wxag054 (PMC13340747; doi:10.1093/annweh/wxag054)
Supplement: wxag054_Supplementary_Data [file wxag054_supplementary_data.pdf]

Title: Perceived physical exertion in senior workers performing heavy manual tasks

Journal name: Annals of Work Exposures and Health

Authors: Albin Stjernbrandt[1], Pontus Öhrner[2], Andreas Tornevi[1], Jerry Öhlin[1], Farhad Abtahi[3,4], Charlotte Lewis[1], Mikael Forsman[3,5], Viktoria Wahlström[1]

Affiliations:

[1]Department of Epidemiology and Global Health, Umeå University, Umeå, Sweden

[2]Sports Medicine, Department of Community Medicine and Rehabilitation, Umeå University, Umeå, Sweden

[3]Division of Ergonomics, KTH Royal Institute of Technology, Stockholm, Sweden

[4]Department of Clinical Science, Intervention and Technology, Karolinska Institutet, Stockholm, Sweden

[5]Institute of Environmental Medicine, Karolinska Institutet, Stockholm, Sweden

Corresponding author:

Albin Stjernbrandt, Associate Professor

Department of Epidemiology and Global Health

Umeå University

901 87 Umeå, Sweden

Tel +46 90 785 99 52

albin.stjernbrandt@umu.se

Table S1. Linear mixed model for Borg's Rating of Perceived Exertion scale among females

Variable	Categories	Estimated coefficient (β)	Ninety-five percent confidence interval		Standard error	*p* value
			Lower	Upper		
Intercept	-	4.84	−3.54	13.23	4.24	0.255
Mean HR (beats/min)	-	0.02	−0.01	0.05	0.02	0.279
Age (years)	-	0.01	−0.08	0.10	0.05	0.860
BMI (kg/m^2)	-	0.08	−0.04	0.19	0.06	0.196
VO_2max (ml/kg/min)	-	0.05	−0.03	0.13	0.04	0.183
Sleep quality	Very good	Reference	-	-	-	-
	Good	0.65	−0.60	1.90	0.63	0.307
	Neither good nor bad	0.54	−0.83	1.92	0.69	0.435
	Poor	0.09	−1.26	1.44	0.68	0.899
	Very poor	3.12	0.95	5.30	1.10	0.005
Psychological stress	1 (Very low)	Reference	-	-	-	-
	2	0.15	−1.83	2.14	1.00	0.878
	3 (Low)	0.47	−0.82	1.77	0.65	0.472
	4	1.60	0.20	2.99	0.71	0.026
	5 (Neither high nor low)	1.26	−0.03	2.54	0.65	0.056
	6	1.90	0.44	3.36	0.74	0.011
	7 (High)	2.61	0.88	4.33	0.87	0.003
Global chronic pain scale – revised	No chronic pain	Reference	-	-	-	-
	Mild chronic pain	−0.79	−1.90	0.33	0.56	0.164
	Bothersome chronic pain	0.95	−0.43	2.33	0.70	0.175
	High impact chronic pain	−0.26	−1.12	0.60	0.43	0.549

HR: heart rate, BMI: body mass index, VO_2max: estimated maximum oxygen consumption. Pseudo-R^2 for final model = 0.306.

Table S2. Linear mixed model for Borg's Rating of Perceived Exertion scale among males

Variable	Categories	Estimated coefficient (β)	Ninety-five percent confidence interval		Standard error	*p* value
			Lower	Upper		
Intercept	-	5.73	−6.46	17.91	6.12	0.352
Mean HR (beats/min)	-	0.04	0.001	0.07	0.02	0.046
Age (years)	-	−0.04	−0.16	0.07	0.06	0.486
BMI (kg/m²)	-	0.05	−0.13	0.23	0.09	0.587
VO_2max (ml/kg/min)	-	0.05	−0.03	0.14	0.04	0.204
Sleep quality	Very good	Reference	-	-	-	-
	Good	−0.23	−1.37	0.91	0.58	0.690
	Neither good nor bad	0.16	−1.07	1.39	0.62	0.800
	Poor	−0.05	−1.50	1.40	0.73	0.943
	Very poor	-	-	-	-	-
Psychological stress	1 (Very low)	Reference	-	-	-	-
	2	2.77	−1.11	6.66	1.95	0.159
	3 (Low)	1.83	0.38	3.28	0.73	0.014
	4	2.12	0.15	4.09	0.99	0.035
	5 (Neither high nor low)	2.45	0.96	3.93	0.75	0.002
	6	2.39	0.56	4.21	0.92	0.011
	7 (High)	1.28	−1.63	4.18	1.46	0.384
Global chronic pain scale – revised	No chronic pain	Reference	-	-	-	-
	Mild chronic pain	−0.20	−1.56	1.17	0.69	0.776
	Bothersome chronic pain	−0.74	−2.75	1.28	1.01	0.469
	High impact chronic pain	0.21	−0.67	1.08	0.44	0.642

HR: heart rate, BMI: body mass index, VO_2max: estimated maximum oxygen consumption. Pseudo-R^2 for final model = 0.243.

Table S3. Linear mixed model for Borg's Rating of Perceived Exertion scale, using relative heart rate reserve instead of mean heart rate.

			**Ninety-five percent confidence interval**			
**Variable**	**Categories**	**Estimated coefficient (β)**	**Lower**	**Upper**	**Standard error**	***p* value**
Intercept	-	8.79	3.31	14.26	2.78	0.002
Relative HRR (%)	-	0.03	−0.001	0.07	0.02	0.058
Age (years)	-	−0.02	−0.09	0.05	0.03	0.524
BMI (kg/m²)	-	0.04	−0.04	0.12	0.04	0.301
VO_2max (ml/kg/min)	-	0.02	−0.02	0.06	0.02	0.225
Sleep quality	Very good	Reference	-	-	-	-
	Good	0.20	−0.57	0.96	0.39	0.616
	Neither good nor bad	0.45	−0.36	1.26	0.41	0.279
	Poor	−0.02	−0.89	0.86	0.45	0.971
	Very poor	3.30	1.47	5.14	0.93	<0.001
Psychological stress	1 (Very low)	Reference	-	-	-	-
	2	1.00	−0.63	2.63	0.83	0.227
	3 (Low)	1.07	0.13	2.00	0.47	0.026
	4	1.89	0.82	2.96	0.54	<0.001
	5 (Neither high nor low)	1.91	0.97	2.84	0.47	<0.001
	6	2.24	1.15	3.33	0.55	<0.001
	7 (High)	2.65	1.25	4.04	0.71	<0.001
Global chronic pain scale – revised	No chronic pain	Reference	-	-	-	-
	Mild chronic pain	−0.39	−1.19	0.41	0.40	0.335
	Bothersome chronic pain	0.63	−0.44	1.71	0.54	0.245
	High impact chronic pain	0.02	−0.57	0.60	0.30	0.957

HRR: heart rate reserve, BMI: body mass index, VO_2max: estimated maximum oxygen consumption. Pseudo-R^2 for final model = 0.243.
